# 

**Published:** 1986-04

**Authors:** 


### Contents

29 **The Imtran Revolution, Telephone Transmission of X-Rays and Scans**
Huw Griffith
30 **An approach to histopathological audit in Bristol**
J. H. F. Smith, S. Z. Al-Sam, J. C. Briggs and J. D. Davies
33 **The CT Diagnosis of Pleural Lipoma**
G. Buirski and P. Goddard
34 **From our Correspondents**
36 **Dr Robert Levet: an obscure practiser in physic**
John Griffiths
38 **Gun Money, owning a piece of History**
Paul Goddard
39 **West Country Physicians Club**
Abstracts of papers
41 **Wessex Branch of the Association of Clinical Pathologists**
Abstracts of papers
44 **Book Reviews**
**Essentials of Dermatology**
J. L. Burton
**The Cuisine of Hungary**
G. Lang
46 **Letters to the Editor**
R. R. M. Harman, Roger Pardoe, and Cameron Kennedy and May Hobby

